# Bridging acute and chronic stress effects on inflammation: protocol for a mixed-methods intensive longitudinal study

**DOI:** 10.1186/s40359-025-02777-y

**Published:** 2025-05-02

**Authors:** Lennart Seizer, Anja Pascher, Sonja Branz, Nadine Schmitt, Johanna Löchner, Björn W. Schuller, Nicolas Rohleder, Tobias J. Renner

**Affiliations:** 1 Department of Clinical Psychology and Psychotherapy for Children and Adolescents, Friedrich-Alexander-Universität Erlangen-Nürnberg, Erlangen, Germany; 2 Department of Child and Adolescent Psychiatry, Psychosomatics and Psychotherapy, University Hospital of Tübingen, Tübingen, Germany; 3 German Center for Mental Health (DZPG), Tübingen, Germany; 4 CHI – Chair of Health Informatics, Technical University of Munich University Hospital, Munich, Germany; 5 GLAM – Group on Language, Audio, & Music, Imperial College London, London, UK; 6 audEERING GmbH, Munich, Germany; 7 Chair of Health Psychology, Department of Psychology, Friedrich-Alexander- Universität Erlangen-Nürnberg, Erlangen, Germany

**Keywords:** Stress, Inflammation, Intensive longitudinal data, Ecologcial momentary assessment, Psychoneuroimmunology

## Abstract

Acute stress triggers adaptive physiological responses—including transient increases in inflammatory cytokines—while chronic stress is associated with sustained inflammatory activity that may underlie the development of various disorders. Despite extensive research on each stress type individually, the transition and interaction between them remain underexplored. This study aims to address this gap by employing an intensive longitudinal measurement burst design. Healthy university students will be recruited and monitored over three one-week assessment bursts, spaced by three-month breaks. Participants will complete ecological momentary assessments four times daily, recording their emotional states, stress experiences, and daily incidents. Simultaneously, saliva samples will be collected at matching time points to measure biomarkers of immune and stress system activity. In addition, daily audio diaries will provide qualitative context through advanced speech analysis techniques. Data will be analyzed using a multi-level modeling approach to differentiate within-person dynamics from between-person variability, accounting for potential moderators. The findings are expected to shed light on how repeated acute stressors transition into chronic stress and how chronic stress burden may influence acute stress responses.

## Introduction

Psychological stress is frequently encountered in everyday life, and ongoing or persistent stress can be a precipitating factor for the onset of diseases affecting both the central nervous system and various bodily organs [1–3]. A potential mediator for the physiological implications of stress is the immune system, specifically through states of inflammation. While temporary spikes in inflammation are essential for survival in the face of physical harm and infection, various studies have shown that social, environmental, and lifestyle factors, including psychological stress, can trigger systemic chronic inflammation [4]. Although our knowledge about the effects of both acute and chronic stress on inflammatory processes has advanced in recent years, there remains a notable deficiency in our comprehension of the transition phase from acute to chronic stress [5]. This project aims to address this gap and enhance our understanding of the temporal dynamics of the link between stress and inflammation.

Regarding acute stress effects, previous research has documented the mechanisms by which stress response systems initiate and modify the functioning of various bodily systems, primarily for immediate adaptation to perceived threats. This includes elevating blood glucose levels, blood pressure, and heart rate, along with triggering an inflammatory response marked by increased levels of inflammatory cytokines, actions largely driven by the autonomic nervous system and the hypothalamic–pituitary–adrenal axis [1]. A variety of acute stressful events have been investigated by previous research in their effects on immune system activity, ranging from brief everyday stressors (e.g., academic exams), over life event stressors (e.g., death of loved ones), to standardized laboratory stressors (e.g., Trier Social Stress Test). Across all these stressors, effects on immune system activity have been found, such as increases in peripheral inflammatory cytokines, such as interleukin (IL)-1β, IL-6, and tumor-necrosis-factor (TNF)-α [6–10]. These changes may be part of an adaptive fight-or-flight response to stressful situations, which evolutionarily carried the risk of injury and infection.

With regard to chronic stress, there is a consistent association between the exposure to adverse psychosocial conditions over extended periods and increased levels of inflammation [2, 10, 11]. Thereby, chronic stress has been studied in various forms like early life stress, caregiving stress, occupational stress, socioeconomic status, and social isolation [12–15]. As noted above, the resulting systemic chronic inflammation has been proposed as a mediator for the development of various diseases [1, 2, 4, 10, 11]. Furthermore, recent research indicates that the increase in inflammatory activity under chronic stress conditions is not uniform; it tends to be more pronounced in those experiencing greater distress and less so in individuals less impacted by their experiences [5]. Similarly, elevated chronic stress burden has been associated with increased acute stress perceptions and blunted cortisol responses to acute stress [16], and early life adversity has been shown to amplify inflammatory responses to stress in later life [17].

Despite substantial evidence on the biological effects of both acute and chronic stress, their interrelation is not as frequently studied together as it should be. The underlying assumption is that individuals will inevitably face stressful situations multiple times, such as workplace stress or interpersonal conflicts, which initially may trigger acute stress responses aiding in coping or survival. Over time, if these stressors recur, they can evolve into chronic stress, leading to prolonged periods of stress exposure, spanning months to years. There is a notable research gap in exploring this transition from acute to chronic stress, particularly in the context of inflammatory responses, raising the questions: When do repeated acute stressors transition to being considered chronic stress? And how does chronic stress burden alter acute stress effects?

## Present study

To answer these questions, we will use an intensive longitudinal study design to face several methodological challenges associated with the investigation of psychoimmune covariances [18–20]: (1) Traditional self-report methods may overlook the subjective nature of stress appraisal, assuming a uniform response to stress across individuals [21, 22]. This can dilute the actual association between stress perceptions and physiological responses, as individual variations in interpreting stress levels are not accounted for. Adopting repeated measurements and a within-person perspective could mitigate this issue by comparing stress levels to an individual’s average state [23]. (2) There is often a mismatch in timing between the assessment of psychological states and biological markers [24–26]. Since these measures often cover different time frames, it becomes difficult to accurately match psychological stress levels with their corresponding physiological responses. (3) Relying on individuals to recall and assess their stress levels over past periods retrospectively introduces various memory biases that compromise the reliability and validity of these measures [27]. (4) Personally meaningful real-life incidents can be much more intense and produce stronger responses (e.g., in cortisol levels and heart rate) compared to laboratory tasks, which casts doubt on the assumption that laboratory results can be generalized to real life [28, 29]. Therefore, we advocate for the use of ecological momentary assessment (EMA) techniques as a solution to these methodological challenges [30]. EMA involves real-time, repeated measurements of an individual’s conditions and states in their natural environment, offering a more accurate and immediate correlation between psychological stress and physiological responses. Further, the data enable a nuanced analysis that can distinguish between within-person effects (how stress levels fluctuate relative to an individual’s norm) and between-person effects (differences across individuals).

The primary objective of this project is to elucidate the relationship and interaction between acute and chronic stress effects on immune system activity. Systemic inflammation is considered a key mediator by which stress exerts its various effects on physical and mental health [4]. Understanding how and when these effects occur and what risk and resilience factors act as moderators will provide the basis for preventive strategies in many applications. The proposed study will allow to answer questions on how characteristics of acute stress responses, such as intensity and duration, vary as a function of chronic stress burden; how the accumulation of acute stress may lead to dysregulation in the stress and immune systems and the transition of acute to chronic stress responses; and how risk and resilience factors at the level of individual stressors (e.g., exposure time of day and stress handling) or at the level of individual humans (e.g., emotion regulation skills and early life adversity) may explain differences in acute and chronic stress reactivity. Additionally, recent works have criticized the use of single-time measurements to determine the level of biological variables in psychiatry, as the variability and stability of these over time is largely unknown [19, 31]. This work will provide data to estimate the variability of some of these variables within and between participants and thus lay a foundation for future works.

## Methods

### Study design

**Fig. 1:**
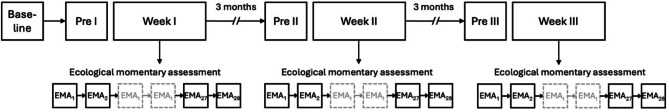
Study timeline

The study follows a measurement burst design [32] with ambulatory psychological and biological assessments in the everyday life of participants (Fig. 1). Assessments will take place over three distinct weeks, each separated by a three-month break. During the assessment weeks, participants will complete EMAs regarding their emotions, stress, and daily incidents four times per day between 10:00 and 22:00 in 4-hour intervals. To lessen the disruptiveness of the study, the participants will have the choice to slightly adjust these times to suit their schedules and the last time point can be brought forward corresponding to the participants bedtime if necessary. At the last time point of each day, the participants additionally record an audio diary (approx. two minutes) in which they report on emotional incidents during the day. Further, at the same times of the EMAs, the participants collect saliva samples for later determination of immune system activity. The samples will be stored refrigerated by the participants and brought to the laboratory afterwards. There, the samples will be stored at -80 °C and analyzed after the collection period is completed to limit batch effects. Before each assessment week, the participants complete an initial baseline questionnaire at the beginning of each week to get information like the life events during the past three months. Further, prior to study start, there will be an introductory onboarding session via an online video-call with each participant to check eligibility criteria and provide them with thorough introduction and explanation of the study procedures. After this session, they will receive the study materials and complete an entry questionnaire.

### Participants

A sample of 80 university students will be recruited using flyers, posters, online ads, and mailing lists. To participate, they must be of legal age, fluent in German, possess a smartphone to complete the online surveys and voice recordings, live within reasonable range of the study side to organize sample transportation, and their daily routine must be compatible with the requirements of the studies, e.g., no night-shift workers. Further, participants must not have current severe immunological or endocrinological diseases or be using immune-modulating drugs. Recruitment is expected to be completed by mid-2026. In previous studies, we have made good experiences with providing participants with feedback reports on their questionnaire and biochemical results, which has helped regarding the recruitment of participants and their compliance with the study protocols [33]. To create and distribute these reports we will use a free-to-use software: Feedback Report for EMA Data [34]. The proposed sample will allow to perform the main statistical analyses with a power above 90% considering an alpha error of 5% and a missing data rate of 20%, which has been found to be the average missing data rate for similar studies [35]. The power analysis was performed using the *simr* package [36] in *R* 4.4 [37] and the specifications reported in [38].

### Questionnaires

All questionnaires will be completed by the participants using the software *REDCap* [39] on their personal smartphones. There are three different sets of questionnaires in this study: (1) the baseline survey that each participant completes before study start, (2) the pre-burst survey that the participants complete on the day prior to the beginning of each assessment week, and (3) the EMA survey that the participants complete at each measurement time point throughout the assessment weeks.

#### Baseline survey

At baseline, prior to study start, the participants complete a self-report questionnaire on general demographics and medical history, the Childhood Trauma Questionnaire [40, 41], the Symptom Checklist-90-R [42, 43], the Big Five Inventory-10 [63] and the Munich Chronotype Questionnaire [44].

#### Pre-burst survey

The day before each EMA week, the participants complete the Trier Inventory of Chronic Stress with a recall interval of three months [45], the UCLA Loneliness Scale [46, 47], the Difficulties in Emotion Regulation Scale [48, 49], the Pittsburgh Sleep Quality Index [50, 51], and the International Physical Activity Questionnaire [52, 53].

#### EMA survey

During the EMA weeks the participants complete these questionnaires four times daily: the Perceived Stress Scale-4 [54, 55], the Multidimensional Mood Questionnaire [56], the Patient Health Questionnaire-4 [57] rephrased to the EMA setting, and a questionnaire on contextual variables related to the saliva sampling. Further, the morning assessment additionally contains items on sleep quality and timing.

### Saliva sampling

In the collection of the saliva samples during everyday life, we follow previous guidelines for best practices [58–60]. During the assessment weeks, saliva will be sampled at four times throughout the day (10:00, 14:00, 18:00, and 22:00). The participants will be instructed to wake up and get out of bed latest by 8:30 during the assessment weeks to avoid distortions of biomarker levels by hormonal awakening responses. Further, they will refrain from brushing their teeth, keep physical activity to a minimum and take nil-by-mouth apart from water during the 30 min prior to each collection time point. Besides the analyte concentrations at each time point, from the samples we are additionally able to determine the total daily output as the area under the curve, the dynamic change as the circadian trajectory, and the average levels per week and per participant. Saliva samples will be collected using cryovials and saliva collection aids. After collection the participants place the samples immediately in a small cool bag (approx. 4 °C), which they receive as part of the study materials, and store them in a freezer at home (approx. -20 °C) as soon as possible. In the samples, analytes of the immune and stress system will be measured that have been found to be stable under these conditions and have been recommended for use in ambulatory assessment studies with low levels of investigator control for protocol compliance (e.g., IL-1β, cortisol, alpha-amylase) [38, 58].

### Audio diary

During the assessment weeks, after completing their questionnaires each evening, participants will record a two-minute spoken diary in which they describe the day’s emotional highs and lows. Hereby, as with the questionnaires, *REDCap* will be used for voice recording and transmission, allowing for unconstrained recording settings. The methodology to analyze these recordings draws upon the integration of automatic speech recognition and natural language processing techniques to perform a comprehensive analysis of both, the content within spoken emotion diaries and paralinguistic markers of the participants’ voice. This approach is motivated by the growing body of research that has successfully applied artificial intelligence to recognize and understand psychological states based on speech data. For example, prior research has shown the emotion of a speaker, the severity of depression, sleepiness, cognitive and physical load can be derived from speech data [61, 62]. Further, content analyses will allow to get contextual information of events that happened throughout the day for interpretation of the EMA and saliva data.

### Data analysis

A multi-level model approach will be utilized to investigate the effect of stress and psychological states on salivary markers over momentary, daily, and weekly timeframes. This approach is well-suited for examining data with nested or clustered structures, where observations are grouped within higher-level units, such as repeated measures within participants. The estimated model can be conceptually divided into four levels. At Level 1, the relationships of the variables at each moment within each day are described, that is for example the momentary stress and momentary cytokine concentrations. At Level 2, the relationship of the day-level variables will be described, for example the average daily stress state and the daily output or circadian profile of cytokines, and further the variability of Level-1 effects are modeled. At Level 3, the relationship of the week-level variables will be described, for example the average weekly stress state and the weekly output of cytokines, and further the variability of Level-1 and Level-2 effects are modeled and may be explained by moderators like the chronic stress burden. At Level 4, the relationship of the time-invariant variables will be described, for example the average stress state and the output of cytokines across the whole study period, and further the variability of Level-1, Level-2, and Level-3 effects are modeled and may be explained by moderators like sex and body-mass index. Multiple random effect structures will be tested to determine which covariates should include random effects. Thereby, a baseline model (random intercept model with fixed effects of all covariates) will be compared to several models, all of which include one additional random effect for one covariate. The model fit will be compared using a likelihood ratio test and the Bayesian information criterion (BIC).

## Discussion

This protocol outlines an intensive longitudinal mixed-methods approach to elucidate the interplay of acute and chronic stress responses with inflammatory states. By combining EMA with repeated salivary biomarker measurements and audio diaries, the study promises a detailed mapping of real-life stress dynamics. The integration of quantitative and qualitative data is expected to reveal subtle interactions between immediate stress reactivity and the cumulative effects of chronic stress, thereby addressing critical gaps in the current understanding of stress-immunity relationships. Moreover, the multi-level modeling approach offers a robust framework to dissect within-person fluctuations and between-person differences. The anticipated findings will not only deepen insights into the psychoneuroimmune mechanisms underlying stress-related disorders but also pave the way for tailored preventative strategies that address both transient and sustained stress exposures.

## Data Availability

No datasets were generated or analysed during the current study.

## Abbreviations

AUC: Area under the curve
BIC: Bayesian information criterion
EMA: Ecological momentary assessment
IL: Interleukin
REDCap: Research electronic data capture
TNF‑α: Tumor necrosis factor‑alpha
